# Examining working mothers’ experience of exclusive breastfeeding in Ghana

**DOI:** 10.1186/s13006-020-00300-0

**Published:** 2020-06-17

**Authors:** Gordon Abekah-Nkrumah, Maame Yaa Antwi, Jacqueline Nkrumah, Fred Yao Gbagbo

**Affiliations:** 1 Department of Public Administration and Health Services Management, University of Ghana Business School, P. O. Box 78, Legon, Accra, Ghana; 2 Department of Health Administration and Education, Faculty of Science Education, University of Education, Winneba, P.O Box 25, Winneba, Central Region Ghana

**Keywords:** Working mothers, Exclusive breastfeeding, Ghana

## Abstract

**Background:**

Although substantial evidence exists on factors that influence exclusive breastfeeding, there is a general lack of qualitative studies that examine how specific workplace factors constrain or promote exclusive breastfeeding among working mothers. The current study therefore examines working mothers’ experience of exclusive breastfeeding, laying emphasis on the influence of workplace factors on working mothers’ decision to exclusively breastfeed their babies.

**Methods:**

The study uses a qualitative research approach and a three-stage purposive sampling procedure to select 20 mothers from 10 organizations in five industries for in-depth interviews on their exclusive breastfeeding experience. Data collected from the interviews were analysed using content analysis, with two major themes emerging for discussion.

**Results:**

The results suggest that two major factors influence exclusive breastfeeding among working mothers: practice of exclusive breast feeding (knowledge and understanding of exclusive breastfeeding, and experience in exclusive breastfeeding) and workplace factors (length of maternity leave, closing time, absence of maternity policy in organizations, inadequate institutional support and family work-life balance).

**Conclusion:**

The results of the study suggest that workplace factors play an equally crucial role in the decision by mothers to exclusively breastfeed their babies. Thus, in the special case of working mothers where breastfeeding prevalence is low, the findings of this study can be crucial in evolving appropriate policies to support working mothers in their effort to exclusively breastfeed their babies.

## Background

Infant nutrition is one of the most important determinants of the health of children, with breastfeeding recommended as the main nutrition for babies. The World Health Organization (WHO) recommends that infants are exclusively breastfed for at least 6 months. In other words, infants receive only breast milk from their mothers or a wet nurse, or expressed breast milk, and no other liquids or solids, with the exception of oral rehydration solution, drops or syrups consisting of vitamins, minerals, supplements or medicines in the first 6 months after birth [1]. There is evidence in the literature to suggest that exclusive breastfeeding has better returns for current and future child health outcomes compared to complimentary food and drinks. For example, exclusive breastfeeding between 6 months and 2 years is suggested to be associated with reduction in infant mortality and morbidity [2–4], lower risk of necrotizing enterocolitis [5], and reduced risk of allergic disease, obesity, type II diabetes, hypertension and hypercholesterolemia in later life [6]. There is also evidence in advanced countries that exclusive breastfeeding protects against gastrointestinal and respiratory infection [5]. These suggest that optimum growth and development can be ensured through exclusive breastfeeding of infants [5]. For mothers, exclusive breastfeeding decreases the chance of developing chronic illnesses related to obesity and the development of ovarian and breast cancer [3], as well as postpartum bleeding [6]. In addition, existing evidence suggests that mothers who exclusively breastfeed their babies are less likely to develop depressive symptoms [7].

Notwithstanding the vast evidence on the benefits of exclusive breastfeeding, the level of exclusive breastfeeding continues to be low both in developed and developing countries. Globally, less than 40% of infants under 6 months of age are exclusively breastfed [8]. Developing countries report an exclusive breastfeeding prevalence of 36% among infants younger than 6 months [9]. However, there is a much lower prevalence of exclusive breastfeeding among professional working mothers in developing countries. A study in Nigeria found the exclusive breastfeeding rate among female doctors to be 11.1% [10]. A recent study in Ghana found the prevalence of exclusive breastfeeding among city-dwelling working mothers to be as low as 10.3% [11]. Working mothers face the challenge of balancing breastfeeding and paid work, thereby increasing the risk of early cessation of breastfeeding in general and exclusive breastfeeding in particular. A key challenge contributing to the early cessation of breastfeeding among working mothers is an inflexible work schedule [12]. In addition, factors such as early return to work, short maternity leave, lack of privacy, feelings of being watched and judged, fatigue and lack of support at work have been suggested to contribute to the low uptake of exclusive breastfeeding among working mothers in China, Kenya and Vietnam [13–15].

While existing studies have been crucial in improving our understanding of the factors that influence the decision by women to exclusively breastfeed, there is a general lack of qualitative studies that examine in detail mothers’ experiences of how specific workplace factors constrain or promote their ability to exclusively breastfeed their babies. Therefore, the objective of this study is to examine the exclusive breastfeeding experiences of working mothers in Accra, the capital city of Ghana. Specifically, the paper aims to:

1. Explore mothers’ exclusive breastfeeding experiences at the workplace.
2. Examine the workplace factors that influence mothers’ decision to adopt exclusive breastfeeding.
3. Assess the challenges working mothers face at the workplace in the practice of exclusive breastfeeding.

The importance of understanding working mothers’ breastfeeding experiences and how workplace dynamics promote or constrain their ability to exclusively breastfeed is based on the fact that working mothers are less likely to exclusively breastfeed compared to non-working mothers [16]. Hence any attempt to understand in detail the factors that promote or constrain exclusive breastfeeding by working mothers will not only be key to further improving the state of the breastfeeding literature, but also evolve appropriate interventions to address the currently low levels of exclusive breastfeeding among mothers in general and working mothers in particular.

**Fig. 1:**
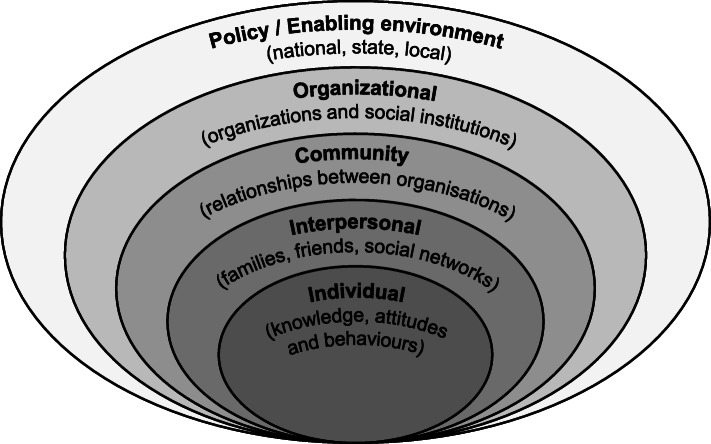
The Socio-Ecological Model**.** Source: Constructed by authors based on Bronfenbrenner, [20]

Several theoretical models and frameworks such as the theory of planned behaviour [17], interactive theory of breastfeeding [18], and Goffman’s theory of social interaction [19], among others, have been used to study the issue of breastfeeding. In the current paper, we use the socio-ecological theory [20] which comprises individual, interpersonal, community, organizational and policy level attributes to explain behaviour. The components of the theory are captured in Fig. 1.

There are five nested, hierarchical levels of the socio-ecological model, starting with the individual level. At the individual level, the characteristics of the individual (reaction, beliefs, attitude, values, goals, etc) shape behaviour and behaviour change process as well as disposition towards a particular health issue. This suggests that the decision to exclusively breastfeed will depend on the attributes of the mother in question. Existing evidence suggests that longer duration of exclusive breastfeeding is significantly associated with positive maternal attitudes toward breastfeeding and good mother-infant bonding [21]. Similarly, mothers’ socio-economic class, education level and mode of delivery have been found to be associated with exclusive breastfeeding practices [4].

The second level of the model is the interpersonal level which captures family, friends, social networks, co-workers and other social support systems that can influence the individual’s behaviour. Thus, we argue that the mother’s willingness to breastfeed exclusively and maintain that for the recommended 6 months will largely be influenced by groups the working mother inter-relates and interacts with. In the context of the current study, co-workers, supervisors and peers at work, in addition to family members, may be critical to the working mothers’ uptake of exclusive breastfeeding. There is evidence in the literature to suggest that adequate family support and higher paternal education are correlated with breastfeeding and exclusive breastfeeding among working mothers, respectively [22].

The third level of the model (community) captures physical and social factors such as household size, parity, family circumstances, partner attitudes and support, and peer support that affect the time, energy and resolve that mothers have for breastfeeding. At the work environment, policies and practices such as working hours, break times, flexibility and baby-friendly or breastfeeding-friendly facilities are critical. In addition, policies that enable on-site expressing and storing of breastmilk influence the ability of mothers to combine work and breastfeeding. For working mothers, this may be an enabler if the conditions are favourable, and vice versa. Hence, exclusive breastfeeding mothers who are working in offices may be successful or not based on prevailing workplace factors. Existing empirical evidence suggests that community level factors as well as the presence of organizational measures aimed at protecting, promoting and supporting breastfeeding are positively correlated with exclusive breastfeeding [23, 24].

The final level captures the policy environment. This is concerned with the formulation, implementation and coordination of policies and/or initiatives supporting exclusive breastfeeding. The argument is that stronger policies that support breastfeeding may produce social mobility in favour of greater practice of breastfeeding [25]. Similarly, if implementation is poor exclusive breastfeeding will not be generally supported and may not be successful. It is therefore important that policy makers appreciate the extent to which the policies they formulate affect exclusive breastfeeding. In this study, policies that cover employees’ rights and privileges are deemed as critical to the successful uptake of exclusive breastfeeding by working mothers. Empirically, public policies such as maternity leave have been found to impact uptake of exclusive breastfeeding. For example, women who had 12 or more weeks of paid leave had increased odds of breastfeeding for six or more months [26]. Additionally, women who returned to work at or after 13 weeks postpartum had higher odds of predominantly breastfeeding beyond 3 months [27].

## Methods

### Study design

The study adopted a qualitative research approach and a purposive sampling procedure to recruit mothers who work in the formal sector to participate in in-depth interviews on their exclusive breastfeeding experience. The study was conducted in Accra, the capital city of Ghana, as it is mostly urban and hosts most of the formal sector businesses in Ghana (ranging from banking, healthcare, manufacturing, hospitality etc).

### Sampling

Professional working mothers in the formal sector in Accra constituted the target population of the study. A three-stage process was used to select participants for the study. In the first stage, sectors (manufacturing, banking, hospitality and healthcare) that are dominant in the business ecosystem in Accra and are easy to reach were selected. In the second stage, two organizations were purposively selected from each industry. In the third stage, eligible respondents were selected from different departments of each of the organizations selected from the second stage. The head of Human Resources at each organization was key in helping the researcher to identify eligible respondents. Women were eligible to participate if they were aged 18 years and above, had given birth to at least one child in the 5 years preceding the interview, had worked in the organization for at least 6 months before the birth of the child in question. On the basis of the above, three and two mothers were respectively recruited from the first and second organization in each industry (see Table 1). The selection of the 20 respondents was considered sufficient to reach information saturation for a qualitative study. The names of the respondents and their organizations have been omitted to maintain anonymity.

**Table 1:** Sociodemographic characteristics of respondents

Industry Represented	Occupation/Rank	Age	Number of Children	Educational Background	Work Experience	No
Manufacturing	Procurement officer	28	2	Graduate	5 yrs.	
	Operations Manager	34	2	MBA	10 yrs.	
	Human resource manager	36	2	Graduate	7 yrs.	
	Personal assistant	31	2	HND	1 yr.	
	Supply chain officer	37	3	Graduate	6 yrs.	5
Banking	Branch manager	29	2	MBA	2 yrs.	
	Bank teller	35	3	Graduate	3 yrs.	
	Bank marketing representative	40	3	MBA	12 yrs.	
	Credit officer	34	4	Graduate	5 yrs.	
	Data processing officer	30	3	MBA	1 yr.	5
Hospitality	General Manager	39	3	Graduate	11 yrs.	
	Waitress	35	3	HND	2 yrs.	
	Event Planner	26	2	HND	1 yr.	
	Chef	32	3	HND	6 yrs.	
	Porter	33	3	HND	1 yr.	5
Healthcare	Pediatrician	34	2	Specialist	6 yrs.	
	Nurse	28	2	Graduate	2 yrs.	
	Pharmacy Technician	30	3	HND	2 yrs.	
	Physician assistant	32	3	Graduate	4 yrs.	
	Health Service Administrator	36	2	MBA	10 yrs.	5
Total						20

*HND* Higher National Diploma, *MBA* Master of Business Administration

### Data collection and analysis

An unstructured interview guide was used to collect relevant data from respondents. The interview guide contained questions on demographic characteristics as well as questions on mothers’ knowledge and experience of exclusive breastfeeding, workplace factors that influence the decision of mothers to adopt exclusive breastfeeding, and challenges that working mothers face at the workplace in their practice of exclusive breastfeeding.

The responses to the interview questions were first transcribed verbatim, and thoroughly read through several times to determine the trend of the responses. The data was analysed manually using content analysis. Content analysis involves reducing and structuring text by identifying, coding, and classifying text into categories [28]. In this study we used the similarities and differences in the respondents’ answers to identify themes related to the research objectives. We identified patterns and characteristics in the data by labelling and classifying the responses relating to each research objective. The basic units of coding were the responses to the interview questions.

Ethical clearance for the study was obtained from the University of Cape Coast Institutional Review Board (IRB) as part of a broader level study on Breastfeeding in Ghana with Ethical Review number UCCIRB/EXT/2018/03. In addition to the ethical clearance, informed consent was sought from respondents after explaining the essence of the study to each respondent.

## Results

### Demographic characteristics of respondents

Table 1 presents the demographic characteristics of the 20 respondents. Six (30%) of the working mothers who exclusively breastfed their babies were aged 20–30 years and the remaining 14 respondents were aged 31–40 years. These results may indicate that Ghanaian working mothers engaged in exclusive breastfeeding range from young to relatively older women. Respondents (five respondents each from health, hospitality, banking and manufacturing sectors) had working experience ranging from one to 12 years. Eight respondents (40%) had two children and 10 respondents (50%) had three children The remaining two respondents had one and four children, respectively.

The collected data were analysed and two themes (practice of exclusive breastfeeding; and workplace factors that either promote or hinder the decision to exclusively breastfeed) emerged. The two themes are discussed below.

### Practice of exclusive breastfeeding

The analysis of the data produced two indicators to examine the practice of exclusive breastfeeding among working mothers: knowledge and understanding of exclusive breastfeeding; and the experience of practicing exclusive breastfeeding among working mothers.

#### Knowledge and understanding of exclusive breastfeeding

The majority of interviewed respondents demonstrated good knowledge of exclusive breastfeeding and acknowledged the important role a six-month period of exclusive breastfeeding plays in the physical and mental development of their babies. The majority alluded to the fact that exclusive breastfeeding meant feeding a newborn child with only breast milk for the first 6 months after birth. A few respondents mentioned that exclusive breastfeeding involves not introducing any food supplements or substitutes in the feeding of a baby for the first 6 months. A respondent from the banking sector explained exclusive breastfeeding to be:

*“Giving the baby only breast milk for the first six months”*

Another respondent mentioned that nursing mothers are required by law to exclusively breastfeed their babies. She explained:

*“I know that, in law, we are supposed to exclusively breastfeed a baby for six months to enable the baby to gain strength.”*

It was further revealed that antenatal sessions served as the greatest source of information for exclusive breastfeeding. Sixteen respondents indicated that their source of knowledge on exclusive breastfeeding began when they started attending antenatal clinics. According to the respondents, education and counselling was given by nurses and midwives. The internet and family members also served as a good source of information. For example, two respondents said the following:

*Respondent 18: “I got the information from the pregnancy school during antenatal visit. Pregnant women are taught the merits of exclusive breastfeeding. I was also taught how to position the baby before breast feeding it.”*

*Respondent 14: “I gathered much knowledge from the internet and got information from my mother because she had given birth before, so had more experience.”*

Finally, when we probed further to understand why the respondents considered exclusive breastfeeding to be important, 14 out of the 20 respondents indicated that exclusively breastfeeding newborn babies was very important for the physical and mental health of both the mother and the baby. In addition, they indicated that they had observed significant improvements in the health and development of their babies and themselves during the period in which they were engaged in exclusive breastfeeding. The respondents suggested that they observed appreciable improvements in the weight of their babies, in addition to better skin pigmentation and pleasant demeanour during the period of exclusive breastfeeding. Some also indicated that exclusively breastfeeding their babies was essential to creating a mother-baby bond. For example:

Respondent 2: *“I realized that my son had put on a lot of weight, his skin was good, and I think generally, his health was very good as compared to the babies of some friends that were not exclusively breastfed.”*

Some respondents also explained that their involvement in exclusive breastfeeding helped them to avoid further pregnancies. A respondent from the health sector gave this succinct response when asked why she found exclusive breastfeeding to be important.

*“Exclusive breastfeeding will prevent you from getting pregnant and thus it will help with the family planning and all that.”*

#### The experience of practicing exclusive breastfeeding among working mothers

Given that all respondents had the experience of breastfeeding at least one child, they understood the importance of exclusive breastfeeding. This notwithstanding, they all faced some challenges in complying with the recommended 6 months of exclusive breastfeeding. All respondents indicated that for the first 4 months (3 months maternity leave and 1 month annual leave) they were physically present to exclusively breastfeed their babies. However, when they returned to work they had to rely on relatives to feed their babies with expressed breast milk in feeding bottles. Also, mothers indicated that while at home (i.e. on leave), they were able to manage their household chores alongside exclusive breastfeeding of their babies. A respondent from the banking sector had this to say about her experience:

*“I didn’t encounter any problems or challenges when I was exclusively breastfeeding at home, because I had a lot of time for the baby.”*

Another respondent explained that:

*“In such cases, I would stop what I am doing and then attend to the baby and breastfeed the baby whenever the need arose. Or at times I check the interval at which the baby takes in the milk and then give to her, and she sleeps for a very long time. When that happens, I get enough time to do other things”.*

The data suggest that mothers had to adopt alternative strategies to enable them to continue the exclusive breastfeeding of their babies, especially after returning to work. A respondent indicated that with the help of her mum who stays home to look after the baby, she fills the feeding bottles with breast milk to last the entire period she will be at work. Additionally, her mom supported her with feeding the baby with breast milk from the bottle at night. This she believed was crucial to enable her to go to work the next day and have enough rest for the production of breast milk. Another respondent averred that she had to let her sister, who was also a breastfeeding mother, to breastfeed her baby when she was at work and resumed feeding the baby with her own breast milk after work:

*“If I were to be at home, I would have been able to breastfeed the baby, but I am not, so my sister who has also given birth, breastfeeds her in my place… I then continue when I get home from work.”*

In one peculiar case, a working mother who lived close by took intermittent breaks from work to go home to breastfeed her baby. The respondent who works in the manufacturing sector said:

*“There are times that the baby’s breastfeeding clashes with work. There are times when you are busy at work and it coincides with the breastfeeding time of the baby. Thus, you try to balance it, as in if you have to breastfeed him at eleven, you try and finish the work at ten-thirty, ten-forty-five, rush home, go and breast feed and come. I had the privilege of living close to the office, so I was doing that after my four months of staying home was up.”*

### Workplace factors

Workplace factors was identified as a major theme that contributes to mothers’ decision to exclusively breastfeed their babies or not. The theme is divided into two sub-themes: workplace factors that promote exclusive breastfeeding; and workplace factors that inhibit exclusive breastfeeding.

#### Workplace factors that promote working mothers’ practice of exclusive breastfeeding

Two major items identified as promoting exclusive breastfeeding were maternity leave and early closing hours for nursing mothers.

#### Maternity leave

The consensus from respondents is that, in general, the workplace environment is not conducive enough for mothers to exclusively breastfeed their babies. This was especially so after their leave period was over. With respect to those workplace factors that influenced mothers’ decision to exclusively breastfeed, it was discovered that the mandatory three-month maternity leave granted to women after they deliver was crucial in the decision for mothers to start exclusive breastfeeding of their babies. Consequently, most mothers thought that the maternity leave period was too short considering that the recommended period of exclusive breastfeeding was 6 months. The statement below from a respondent confirms this.

“*We were given three months to stay at home before you resume - that is the maternity leave. Thus, you can breastfeed your child for that length of time.”*

Respondents stressed that organizational arrangements are usually made to allow mothers to take their annual leave period as an extension of the maternity leave, to enable them to continue with exclusive breastfeeding:

*“The policy says we have three months leave. But we normally add the accumulated annual leave.”*

Some respondents also indicated that in the case of having twins, they were given an additional 2 months and 3 weeks to their mandatory maternity leave period as stipulated by the Labour Law (Act 225), even though they were of the view that this extension was still inadequate. The below response supports this claim:

“*No, we don’t have any policy, we go by the Labour Act. Then after the twins I was given only two months, three weeks. You know twins are such a handful.”*

#### Early closing hours

Aside from maternity leave, organizations allowing breastfeeding mothers to leave before the close of work was essential in helping mothers to continue exclusive breastfeeding. Some of the respondents who admitted to workplace factors serving as enablers said it helped in their decision to exclusively breastfeed. It was discovered that their organizations usually allowed mothers to close an hour or two before the stipulated closing time in order to get back home to breastfeed their babies. The following quotes capture this assertion:

Respondent 9: *“The only thing that helped with that was we closed one hour before the normal closing time. That made it quite easy for me to quickly go home and continue breastfeeding, since I would have expressed enough for him before leaving for work.”*

Respondent 10: *“In the work that we normally do, we close early as breastfeeding mothers. I think we close two hours before everyone does.”*

#### Workplace factors that hinder working mothers’ practice of exclusive breastfeeding

Three sub-items were identified under this sub-theme. They include: absence of maternity policy in organizations; inadequate institutional support; and work-family imbalance.

#### Absence of maternity policy in organizations

Thirteen respondents indicated that the absence of a functioning maternity policy in their organizations had inhibited their ability to “freely” engage in exclusive breastfeeding at the workplace. Most respondents asserted that the 3 month mandatory maternity leave stipulated by law was not enough to encourage breastfeeding mothers to engage in the recommended six-months of exclusive breastfeeding. They were therefore expecting that the mandatory maternity leave could be complemented by an organizational-sponsored maternity leave. Respondents were quick to indicate that the absence of an organizational maternity leave policy had an adverse effect on their practice of exclusive breastfeeding. For example, a respondent indicated:

*“No, we do not have any policy like that. We just go by the Labour Law, that allows for three months mandatory maternity leave and sometimes you are allowed to add your annual leave to it. But to say we have a maternity policy, no.”*

Thus, many breastfeeding mothers are either forced to “steal” time off work to go home at regular intervals to breastfeed their babies or wake up very early to breastfeed and pump breast milk, that is later used to feed their babies when they are at work. The challenge with the former is the huge cost burden that comes with going home frequently to breastfeed. In terms of the latter, breastfeeding mothers end up being very tired, due to sleep deprivation as well as their babies refusing the feeding bottle or the expressed milk not being enough for the baby. This was confirmed by the views of two respondents below:

Respondent 18: *“I go to work at 7:00am and I get home by 3:00pm. But I have to wake up early to use a breast pump to express the milk. That means less sleep. So, I express it into a bottle, then put it in the fridge, and when it’s time, they will feed the baby with it. However, there have been instances where I will be at work and they will call me that the breast milk is finished, or the baby is not taking the milk. You know, when they call you, you have to double up, pick a car and then rush home to feed him. It’s quite difficult.”*

Respondent 6: *“My work is somehow tedious already. I take some work to the house. Then have to juggle house chores with office work and the baby. I settle in around 9:00pm and squeeze breast milk before I sleep. Do it again around 12:00am, and then 4:00am, just to get enough. That is, if baby does not demand for it at the time.”*

As a result of this challenge, the respondents argued that adoption of a maternity leave policy by their respective organizations was fundamental, given that it will allow breastfeeding mothers a longer maternity leave period to complement the mandatory maternity leave. This is adequately captured by the two responses below:

Respondent 5: *“He does not always want to be with me, because I am not the one who normally takes care of him, my mom does. I feel bad about it..*

Respondent 10: *“In my opinion, if I were to be asked whether mothers should be given six months maternity leave, so that they can constantly be by their baby to breastfeed without having to pump into bottles, so that after six months they start work? – I would say yes.”*

#### Inadequate institutional support

The analysis also suggests that lack of institutional support from organizations is one of the factors militating against the smooth practice of exclusive breastfeeding among working mothers. Eighteen of the respondents indicated that apart from there being no functional maternity policy, their organizations were not doing enough to support them to continue exclusive breastfeeding after they had resumed work. Respondents argued that the lack of breastfeeding rooms, breastfeeding breaks and flexible work hours are a few of the factors that constrained the practice of exclusive breastfeeding. When asked whether her organization (banking sector) had made any arrangement for breastfeeding mothers in the work premises, one respondent revealed:

*“No, at our workplace, there is nothing like that. However, I know some organizations (branches of Barclays) that have a rest room for breastfeeding. There you can go and breastfeed. Where I am currently, there is nothing like that.”*

Breastfeeding mothers therefore have no choice than to leave their children at home. A situation that results in many of them not concentrating fully at work. As seen in this response:

*“Yeah, I was leaving him. But I had no choice, I had to leave him and go to work. So, it wasn’t easy. I will be down throughout. When I went to work, I was still thinking of my baby. What is he going through, so I was just making calls throughout, every hour. Asking whether he was okay? Was he asleep? Had he taken the expressed breast milk? I wasn’t comfortable but I had no choice.”*

#### Work-family imbalance

One respondent mentioned the issue of work and family life imbalances as another factor that hinders working mothers’ ability to exclusively breastfeed their babies. For this respondent, her husband also competed with the baby for the breast and this posed a challenge in trying to juggle the two. It was also revealed that the seeming lack of balance between work and family life was having an impact on breastfeeding mothers’ physical health. Many respondents complained that because of their inability to breastfeed their babies due to being at work, they experienced a lot of pain in their breast. This they believe was due to their breast becoming engorged. These sentiments are captured in the two quotes below:

Respondent 3: *“…in my case, because my husband and the baby compete for the breast milk, it left me tired sometimes, making me want to put an end to exclusive breastfeeding.”*

Respondent 5: *“It is very painful and sometimes it seeps out. That’s why I use the breast pad. At work, I can’t do anything, I feel the pain. When I get home, he doesn’t suck, especially when I keep long at work. Thus, I wait for daddy to come, and suck it.”*

## Discussion

The paper set out to qualitatively examine working mothers’ experience of exclusive breastfeeding. Consistent with the socio-ecological theory, the results of the paper suggest that individual, interpersonal, community, organizational and policy level attributes explain mothers exclusive breastfeeding behaviour [20].

First and foremost, the theory explains how mothers’ beliefs and attitudes affect their decision to breastfeed. As per the results, many working mothers’ decision to continue exclusive breastfeeding is in part influenced by the knowledge that breastfeeding the baby helps to give the baby some added advantage compared to non-breastfed babies [4]. Furthermore, existing evidence suggests that a longer duration of exclusive breastfeeding is significantly associated with positive maternal attitudes toward breastfeeding and good mother-infant bonding [21]. The level of mothers education is also associated with the level of dedication in the practice of exclusive breastfeeding [29]. It is common knowledge that the educational level of mothers helps them to make informed decisions on the benefits of exclusive breastfeeding.

The important role of social and emotional support from family in working mothers’ practice of exclusive breastfeeding was again confirmed in this study. Working mothers relied on their close relatives to attend to their babies when they were away at work. This confirms previous findings [29]. It is possible that close relatives and substantial others help mothers to overcome breastfeeding challenges. As a result, mothers who enjoy healthy support from family were able to create a good balance between work and the practice of exclusive breastfeeding. This finding is in line with the second dimension of the socio-ecological theory (interpersonal level), which captures the role and significance of family, friends, social networks, co-workers and other social support systems on breastfeeding mothers’ behaviour. The finding further corroborates Bronfenbrenner’s [20] theoretical argument that mothers’ willingness to breastfeed exclusively for the recommended 6 months will largely be influenced by those groups who the mother inter-relates and interacts with. In the context of the current study, it was found that support afforded to breastfeeding working mothers is critical to the continued practice of exclusive breastfeeding. Study findings further suggest that it helped mothers to be physically and emotionally sound and to exude positive physiological changes in their body that would allow for the production of more breast milk.

The findings of the study equally suggest that other environmental and policy level factors serve as a major challenge affecting working mothers’ ability to practice exclusive breastfeeding. Chief among these factors are inadequate maternity leave duration, and lack of maternity policies and facilities that support breastfeeding at the workplace. For women practicing exclusive breastfeeding, these challenges can result in a surge in anxiety, which as noted by Ismail et al., [30], normally leads to a reduction in breast milk production. In addition, the unavailability of crèches and nursing rooms at the workplace leads to work-family imbalance that ultimately affects mother and child bonding, which is very crucial at the formative stages of the baby’s development. Labbok et al. [25] maintained that these resultant problems provide a strong argument for stronger policies that support breastfeeding. Similarly, Mirkovic et al. [26] argue that even with the availability of these policies, if implementation is poor, the end result will be that exclusive breastfeeding will not be generally supported and may not be successful. It is therefore important that policy makers always appreciate the extent to which the policies they formulate affect exclusive breastfeeding. It was confirmed in this study that policies that cover employees’ rights and privileges such as extended maternity leave, among others, are critical to the successful uptake of exclusive breastfeeding by working mothers.

## Conclusion

The study sought to examine working mothers’ experience of exclusive breastfeeding in Ghana. Consistent with the ecological theory, the results suggest that individual, interpersonal, community, organizational and policy level attributes explain working mothers’ decision to exclusively breastfeed for 6 months. Specifically, knowledge and experience and workplace factors constitute key drivers of exclusive breastfeeding by mothers. This finding is contrary to the constant emphasis on individual level factors such as information dissemination and education of nursing mothers, both within the literature and at the policy level. It is therefore important that policy interventions begin to focus on addressing workplace factors. Through the appropriate incentive system, the state can encourage employers to address issues related to closing time for nursing mothers, provision of institutional support with respect to maternity leave policy in organizations, and work-family imbalance. Addressing these challenges will not only help in promoting exclusive breastfeeding with its intended benefits but will also be instrumental in helping breastfeeding mothers to be productive at the workplace.

It is important to emphasise that drawing a sample of 20 nursing mothers from five sectors in Accra alone may not be enough to generalise the results across Ghana. Notwithstanding, the results remain valid and chances are that extra interviews in other regions or industries will not yield findings entirely different from the current results.

## Data Availability

The data used for the study is available and can be requested from the corresponding author upon reasonable request.
